# XPS Study on Calcining Mixtures of Brucite with Titania

**DOI:** 10.3390/ma15093117

**Published:** 2022-04-26

**Authors:** Karla Sofía Sánchez-Zambrano, Marina Hernández-Reséndiz, Cristian Gómez-Rodríguez, Linda Viviana García-Quiñonez, Josué Amilcar Aguilar-Martínez, Edén Amaral Rodríguez-Castellanos, Luis Felipe Verdeja, Daniel Fernández-González, Guadalupe Alan Castillo-Rodríguez

**Affiliations:** 1 Facultad de Ingeniería Mecánica y Eléctrica (FIME), Universidad Autónoma de Nuevo León (UANL), San Nicolás de los Garza 66450, Mexico; karla.sanchezza@uanl.edu.mx (K.S.S.-Z.); marina.hernandezrsn@uanl.edu.mx (M.H.-R.); josue.aguilarmrt@uanl.edu.mx (J.A.A.-M.); eden.rodriguezcs@uanl.edu.mx (E.A.R.-C.); 2 Faculty of Engineering, University of Veracruz, Coatzacoalcos 96535, Mexico; crisgomez@uv.mx; 3 Departamento de Ciencia de los Materiales e Ingeniería Metalúrgica, Escuela de Minas, Energía y Materiales, Universidad de Oviedo, 33004 Oviedo, Asturias, Spain; lfv@uniovi.es; 4 CONACYT-Centro de Investigación Científica y de Educación Superior de Ensenada B.C. (CICESE), Unidad Monterrey, Apodaca 66629, Mexico; adnilanavivi_1984@hotmail.com; 5 Centro de Investigación en Nanomateriales y Nanotecnología (CINN), Consejo Superior de Investigaciones Científicas (CSIC), Universidad de Oviedo (UO), Principado de Asturias (PA), Avda. de la Vega, 4–6, 33940 San Martín del Rey Aurelio, Asturias, Spain; d.fernandez@cinn.es

**Keywords:** brucite, magnesia, titania, Mg(OH)_2_, MgO, TiO_2_, XPS, X-RD, refractories, ceramics

## Abstract

In this work, we studied the phases in a Mg-Ti-O system using a 1:1 formulation of MgO:TiO_2_, mixing synthetic brucite of Mexican origin with TiO_2_ microparticles of high purity, with a heat treatment at 1100 °C for 1 h. Due to its valence electrons, TiO_2_ can contribute to the sintering process to improve density in MgO products. The raw materials and formulation by XPS and X-RD techniques were characterized. The results demonstrate the presence of different oxidation states in titania and the formation of different oxides in the Mg-Ti-O system when mixed and calcined at 1100 °C; additionally, we estimated the formation of vacancies in the crystal lattice during the transformation from hexagonal brucite to magnesia with a cubic structure centered on the faces. Its thermal behavior is indicated by the MgO-TiO_2_ phase diagram.

## 1. Introduction

Sintered MgO is one of the most important ceramic materials for the manufacture of basic refractory products; it has been used for many years in the production of steel, cement, and many other products on an industrial scale [1]. One of the sources of raw material to obtain sintered MgO is brine [1,2]; 14% of the world’s MgO production is synthetic magnesia and comes from the precipitation of magnesium hydroxide from seawater sources and brines. In Mexico, MgO is produced in the form of hydroxide, caustic, sintered, and melted from the precipitation of brine combined with calcined dolomite. The initial material for obtaining sintered magnesia is synthetic Mg(OH)_2_, which is precipitated from brine combined with doloma; thus, in the form of impurities, this material contains other oxides from the composition of the brine, mainly from dolomite, for example, SiO_2_, CaO, Fe_2_O_3_, and Al_2_O_3_ [2].

Magnesium hydroxide is a chemically defined compound with hexagonal/rhombohedral crystal structure; the product obtained during thermal decomposition at 1100 °C, caustic MgO, occurs in a cubic crystalline transformation centered on the faces [2,3]. During the thermal decomposition of magnesium hydroxide and crystallographic transformation, the presence of impurity ions in the brucite from dolomite can be an influence; this influence finally impacts the last stage to sinter and obtain the sintered MgO [2]. Regarding the above, it is feasible to incorporate different ions into the crystal lattice of the base material, mainly in the form of microparticles in brucite, and evaluate their influence on properties such as the melting point of MgO [4]. It is feasible that the addition of Ti^4+^ cations modifies the structure of raw materials for MgO-based refractories when they are added to Mg(OH)_2_ brucite before calcination at low a temperature for hydroxylation. It is intended to demonstrate how the addition of Ti^4+^ cations added to brucite and their calcination at low a temperature modifies the conditions of caustic MgO for the manufacture of raw materials for MgO-based refractories [4].

The novelty of this study is the focus on obtaining a microstructural study and surface composition analysis to determine and compare the chemical oxidation states of the generated phases by determining bond energies prior to the sintering treatment. To obtain high-density, industrial-grade MgO from synthetic brines, the brucite is first calcined at a relatively high temperature to obtain caustic MgO, and then compacted and calcined at a high temperature to obtain dense MgO. There are many studies on the addition of oxides to caustic MgO to obtain magnesia with better density, but there are no references on studies adding oxides to the brucite prior to the step of obtaining caustic MgO. In this way, the current work highlights the importance and novelty of adding micro-TiO_2_ in brucite and opens the possibility of new work in the future, using this route of preparation of MgO-micro-oxide composites to evaluate other physical, mechanical, and chemical properties of the compounds obtained.

In this work, prior to the sintering of MgO, Mg^2+^ cations were replaced by Ti^4+^ cations during the calcination of brucite; this substitution is expected to generate crystalline imperfections in the MgO as vacancies due to differences in ionic radius and valence number. This work contributes to the knowledge of the formation of dense refractory ceramic phases that can occur when we add TiO_2_ to brucite during the manufacture of dense MgO, as well as its possible implications for performance during its application in industrial furnaces at a high temperature, since one of the main problems of the refractory industry is obtaining refractory bricks with dense phases.

We used X-ray photoelectron spectroscopy (XPS) and X-ray diffraction (XRD) techniques. Through these techniques, we analyzed and compared the chemical states of the elements involved, determining the specific binding energies of each emitting element, elementary composition, and generation of new phases.

Other researchers have also studied MgO composites using the XPS technique [5]. Garcia et al. studied sintered MgO with the addition of nanoparticles in different percentages by weight (iron oxide and aluminum oxide) using the XPS technique, to determine the oxidation states generated after laser irradiation with different wavelengths (532 and 1064 nm). The results showed that the binding energies varied according to the wavelength, energy fluence, and concentration of nanoparticles used after the samples were irradiated [6,7]. In another work, the influence of the preparation procedure of MgO nanopowders on the surface properties was investigated by XPS. The powders were obtained via two methods: (1) by gelation/precipitation from a solution containing Mg^2+^, and (2) by a micellar liquid which was created by adding the Mg^2+^ solution to a surfactant. The XPS analysis showed the presence of magnesium carbonate in the outermost layers, and the presence of Mg(OH)_2_ was evident [8]. Sun et al. prepared Mn_2_CoAl thin films on MgO substrates by magnetron sputtering and heat treatment at 300 °C. They used the XPS technique to study the evolution of the chemical states of Mn_2_CoAl/MgO after annealing. The reported binding energy for MgO was 1303.80 eV, and there were changes in binding energies, indicating Mg oxidation at the interface [9].

Based on the above, in this work, the phases in the Mg-Ti-O system were studied using a 1:1 formulation of MgO:TiO_2_, mixing synthetic brucite of Mexican origin with high purity TiO_2_ microparticles, with a heat treatment at 1100 °C for 1 h. The raw materials and formulation were characterized by XPS and XRD techniques. The results demonstrate the presence of different oxidation states in titania and the formation of different oxides in the Mg-Ti-O and Ca-Ti-O systems when mixed and calcined at 1100 °C; additionally, we estimated the formation of vacancies in the crystal lattice during the transformation from hexagonal brucite to magnesia with a cubic structure centered on the faces. Its thermal behavior is indicated by an MgO-TiO_2_ phase diagram produced.

## 2. Materials and Methods

### 2.1. Materials

The magnesia used in this work is high purity and industrial grade, produced in México from brines with the addition of doloma by Grupo Peñoles company (Laguna del Rey, Coahuila, Mexico). Although there are different production methods for obtaining MgO [2,10], the following explains how MgO is obtained in Mexico, starting from dolomite. Doloma is obtained from the calcination of dolomite, which provides 40% of the final magnesium ions. The doloma is mixed with MgCl_2_ salts in aqueous solution obtained by crystallization from a natural brine mantle, according to the following chemical reaction:

(1) $${\mathrm{MgSO}}_{4}\left(\mathrm{ac}\right)+2\mathrm{NaCl}\left(\mathrm{ac}\right)\to {\mathrm{Na}}_{2}{\mathrm{SO}}_{4}↓+{\mathrm{MgCl}}_{2}\left(\mathrm{ac}\right)$$

The aqueous solution of MgCl_2_ is mixed with the doloma at room temperature, producing the following consecutive chemical reactions:

(2) $$\mathrm{MgO}\cdot \mathrm{CaO}+H_{2}O\to \mathrm{Mg}{\left(\mathrm{OH}\right)}_{2}↓+\mathrm{Ca}{\left(\mathrm{OH}\right)}_{2}\left(\mathrm{ac}\right)$$

(3) $$\mathrm{Ca}{\left(\mathrm{OH}\right)}_{2}\left(\mathrm{ac}\right)+{\mathrm{MgCl}}_{2}\left(\mathrm{ac}\right)\to \mathrm{Mg}{\left(\mathrm{OH}\right)}_{2}↓+{\mathrm{CaCl}}_{2}\left(\mathrm{ac}\right)$$

The aqueous CaCl_2_ is treated with the depleted brine of reaction (1), which results in more magnesium ions:

(4) $${\mathrm{MgSO}}_{4}\left(\mathrm{ac}\right)+{\mathrm{CaCl}}_{2}\left(\mathrm{ac}\right)+2H_{2}O\to {\mathrm{MgCl}}_{2}\left(\mathrm{ac}\right)+{\mathrm{CaSO}}_{4}2H_{2}O↓$$

**Figure 1:**
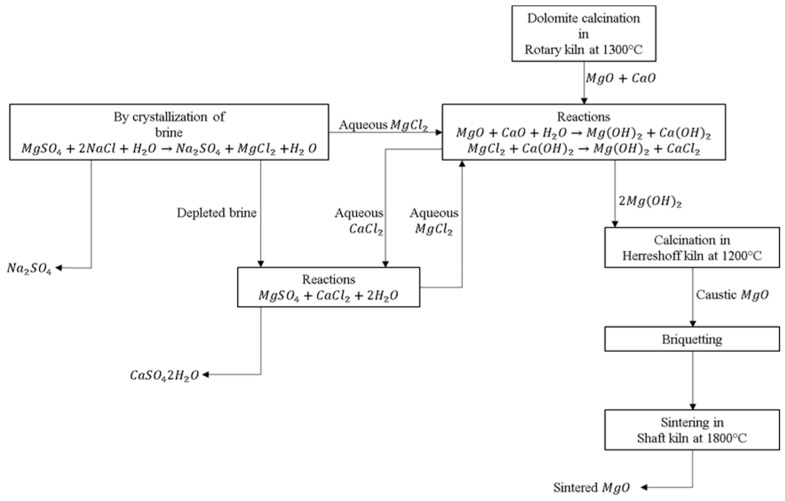
Representative diagram of the process for obtaining caustic MgO in Mexico [11].

The aqueous MgCl_2_ is subsequently treated with reaction (3) to obtain more magnesium ions. The magnesium hydroxide obtained from reactions (2) and (3) is calcined in a multi-home Herreshoff furnace at 1100 °C, and the industrial grade caustic MgO is finally obtained with 99.9% purity. The reactions of the process are outlined in Figure 1.

(5) $$\mathrm{Mg}{\left(\mathrm{OH}\right)}_{2}\overset{\Delta }{\to }\mathrm{MgO}+H_{2}O↑$$

The brucite obtained has a chemical composition as shown in Table 1, with high MgO content; loss on ignition (LOI) corresponds to the high content of chemical water in the form of ions (OH)^2−^ associated with Mg and the content of water in physical form from the brine solution.

**Figure 2:**
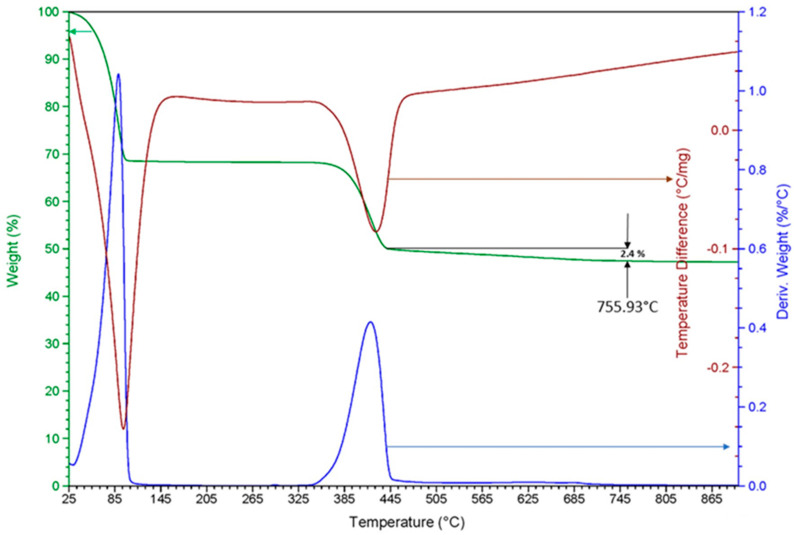
Results of the thermogravimetric and differential thermal analysis performed on brucite.

A thermogravimetric analysis of brucite reveals that physical water mass loss and chemical water loss (dehydration) occur at temperatures of 105.01 °C and 450.3 °C, respectively (Figure 2). The loss of mass at 755.93 °C is due to the loss of residual chlorides from synthetic brine. The weight loss corresponds to 2.4%.

Titania of high purity (99.99%) from Sigma Aldrich, in the form of microparticle powder, was used as an additive in the development of the present work. For sample preparation, the chemical reactions expected during the thermal process are as follows:

(6) $$\mathrm{Mg}{\left(\mathrm{OH}\right)}_{2}\overset{\Delta }{\to }\mathrm{MgO}+H_{2}O↑$$

(7) $$\mathrm{MgO}+{\mathrm{TiO}}_{2}\overset{\Delta }{\to }{\mathrm{MgTiO}}_{3}$$

That is, the expected complete reaction is as follows:

(8) $$\mathrm{Mg}{\left(\mathrm{OH}\right)}_{2}+{\mathrm{TiO}}_{2}\overset{\Delta }{\to }{\mathrm{MgTiO}}_{3}+H_{2}O↑$$

Table 2 shows the percentages by weight of the amount of brucite used in the formulations of the present work, based on the molecular weights of the original substances and the product of the expected reaction.

### 2.2. Sample Preparation

Weight percentages of TiO_2_ microparticles were added to Mg(OH)_2_ powders considering the following relation: (100 − X) wt.% Mg(OH)_2_ + X wt.% of TiO_2_, where X = 0, 100, and 50. The formulations studied in this work are presented in Table 3.

For the preparation of the mixtures, TiO_2_ and brucite were mixed; the brucite was mixed with the titania in a porcelain mortar and homogenized manually. Subsequently, the mixture was placed in high-alumina crucibles and placed in an oven, where they were calcined at a maximum temperature of 1100 °C for 1 h. Finally, the powder samples of caustic MgO mixed with TiO_2_ particles were obtained. The mixtures were calcined out in a Lindberg Blue M/1700 Thermo Fisher Scientific electric furnace (Facultad de Ingeniería Mecánica y Eléctrica, UANL, San Nicolás de los Garza, Nuevo León, Mexico), using a heating rate of 5 °C/min and a dwell time of 1 h at maximum temperature. Cooling down to room temperature was carried out in the furnace.

### 2.3. Methods

#### 2.3.1. Characterization by Spectrometry of X-ray-Induced Photoelectrons (XPS)

The samples were placed on carbon-conductive tapes to perform X-ray-Induced Photoelectron Spectroscopy (XPS) analysis on Thermo Scientific Inc. Model K-Alpha equipment (Facultad de Ingeniería Mecánica y Eléctrica, UANL, San Nicolás de los Garza, Nuevo León, Mexico). This analysis was performed with a monochromatic Al K radiation with energy E = 1486.68 eV.

Cleaning by a soft surface etching step was performed to remove superficial impurities from the sample during the analysis. Binding energies of all the peaks were corrected using C 1 s energy at 284.6 eV, corresponding to adventitious carbon. Moreover, the charge compensation was corrected by the flood gun associated with the spectrometer. The peaks were deconvoluted using a Shirley-type background calculation and peak fitting using the Gaussian–Lorentzian sum function.

#### 2.3.2. X-ray Diffraction Characterization

X-ray diffraction characterization was performed with a Panalytical Empyrean model diffractometer, with Co radiation with a wavelength of 1.79 Å (Facultad de Ingeniería Mecánica y Eléctrica, UANL, San Nicolás de los Garza, Nuevo León, Mexico). The samples were analyzed with a scanning range of 10 to 144° at a scan speed of 1°/s, using a voltage of 40 Kv and current of 40 mA, to investigate the crystallographic information. Data analysis and the peak profile fitting were carried out using the XPowder program.

## 3. Results and Discussion

### 3.1. Analysis of Chemical State by XPS

**Figure 3:**
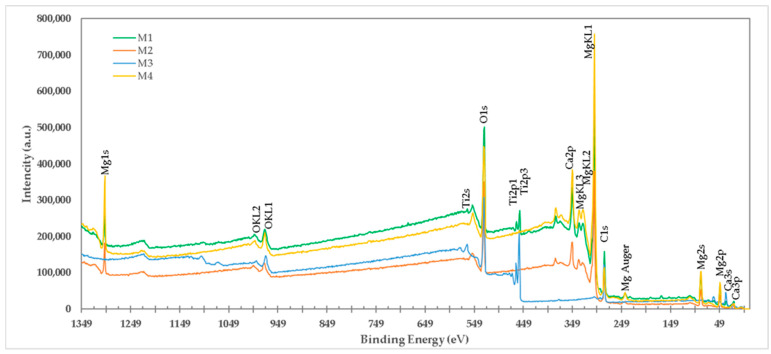
High-resolution spectrum of XPS of M1, M2, M3, and M4 formulations. For the M1 formulation (Brucite + TiO_2_ treated at 1100 °C 1 h with final ratio 1:1 Molar of MgO:TiO_2_), the presence of ions of Mg, Ti, Ca, and O was detected. For the M2 (calcined brucite at 1100 °C for 1 h) and M4 formulations (uncalcined brucite), the presence of Mg, Ca, and O ions was detected. For the M3 formulation (TiO_2_), the presence of Ti and O ions was detected.

For all experiments, the electron bonding energy in carbon was adjusted to 284.6 eV; this is suggested to be a carbon pollutant on the samples due to their handling. The XPS technique provides information on the change in the chemical status [12] of the species that make up the mixtures. In this work, variations in the chemical states of “O”, “Mg”, “Ca”, and “Ti” in the different samples obtained were analyzed. Figure 3 shows the spectra obtained by XPS from the formulations M1, M4, M2, and M3. The intensities of the peaks of O1s and Ti2p decrease when TiO_2_ is added to the brucite, indicating a decrease in these chemical states with the addition of titania and after the treatment of calcination of the samples at 1100 °C for 1 h.

**Figure 4:**
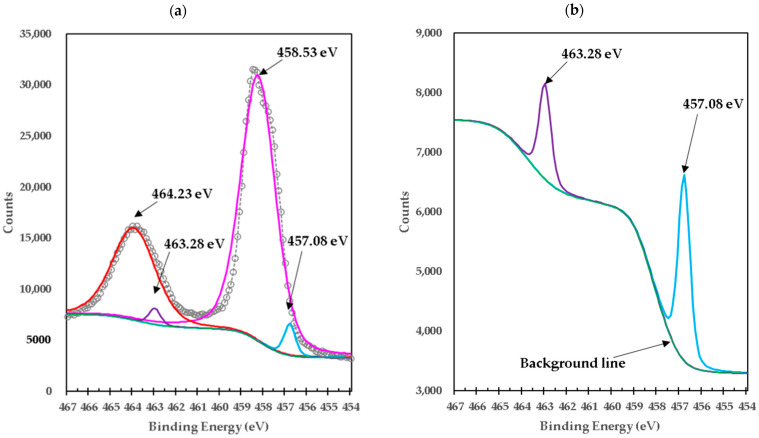
(**a**) High-resolution XPS spectra of Ti2p in high-purity titania; (**b**) details for sample M3.

Figure 4a shows the deconvoluted high-resolution XPS spectrum of Ti in pure TiO_2_. In this spectrum, the Ti2p_3/2_ doublet with binding energy 458.53 eV and Ti2p_1/2_ with binding energy 464.23 eV arises from the division of the spin orbit. These results are consistent with Ti^4+^ and are the characteristic features of the TiO_2_ crystallographic structure [13,14,15]. Furthermore, the calculated difference in binding energy (BE) of Ti2p_3/2_ and Ti2p_1/2_ (∆BE = BE Ti2p_3/2_ − Ti2p_1/2_) was equal to 5.7 eV, which can be assigned to the typical Ti^4+^–O bonds in TiO_2_. The 2p doublet peaks after deconvolution exhibited a tail in the region of lower binding energy, indicating the presence of lower Ti valence states, observed at the peak at a binding energy of 457.08 eV (Ti2p_3/2_) and 463.28 eV (Ti2p_1/2_) corresponding to Ti^3+^ in the Ti_2_O_3_ lattice [12] (details are shown in Figure 4b). This means that both TiO_2_ and Ti_2_O_3_ are present in pure titania. The existence of Ti^3+^ in TiO_2_ indicates that oxygen vacancies are generated to maintain electrostatic equilibrium according to the following chemical equation:

(9) $$4{\mathrm{Ti}}^{4+}+O^{2-}\to 4{\mathrm{Ti}}^{4+}+2e^{\prime \prime }/□+0.5O_{2}\to 2{\mathrm{Ti}}^{4+}+2{\mathrm{Ti}}^{3+}+□+0.5O_{2}$$

The □ represents an empty position that originates from the removal of O^2−^ from the crystalline structure. From the equation, it can be deduced that a generated vacancy of oxygen is accompanied by two Ti^3+^ ions. Therefore, with the areas obtained at each peak of binding energy by XPS, it is feasible to determine the percentage of vacancies of O with the following equations [16]:

(10) $$\begin{array}{c} {\%\mathrm{Ti}}^{3+}={\mathrm{Ti}}^{3+}/{\mathrm{Ti}}^{4+}={\mathrm{area}\;\mathrm{Ti}}^{3+}/{\mathrm{area}\;\mathrm{Ti}}^{4+}\\ {\%\mathrm{Ti}}^{4+}=1-{\mathrm{Ti}}^{3+}\\ O/\mathrm{Ti}=2{\;\mathrm{in}\;\mathrm{TiO}}_{2}\\ O/\mathrm{Ti}=2{\%\mathrm{Ti}}^{4+}+\frac{3}{2}{\%\mathrm{Ti}}^{3+}\\ \%O=\frac{O/\mathrm{Ti}}{2}\\ \%\;\mathrm{Vacancies}\;O=1-\%O \end{array}$$

We calculate that the Ti^3+^/Ti^4+^ ratio yields approximately 6% of the peak areas, and the percentage of oxygen vacancies in the high-purity titania crystalline structure used in this work is 2%. Table 4 presents the data obtained from the measurements of pure titania by XPS and the calculations to obtain the percentage of oxygen vacancies in the crystalline structure.

**Figure 5:**
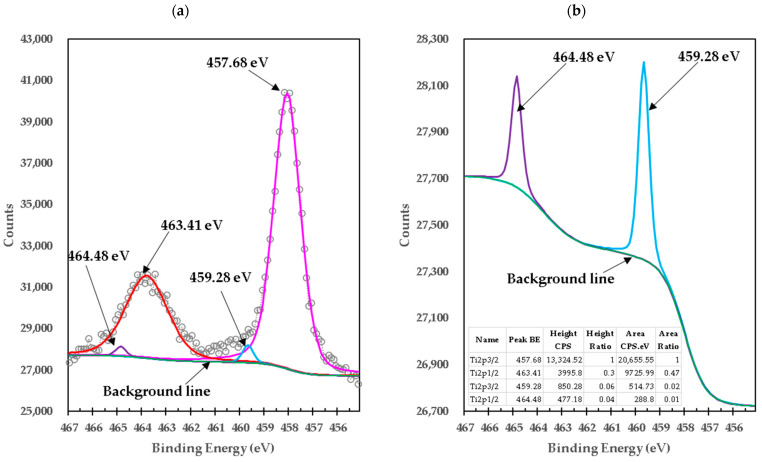
(**a**) XPS high-resolution spectra of Ti2p from TiO_2_ mixed with brucite and calcined at 1100 °C for 1 h; (**b**) details for sample M1.

After mixing brucite with titania and a treatment at 1100 °C for 1 h, the deconvoluted high-resolution XPS spectrum in Figure 5 shows a slight change in position along with a variation in area of the peaks with respect to those of pure titania, showing a negative shift of 0.56 eV (Figure 5). The peaks in the mixed samples of brucite with TiO_2_ are now at the binding energies 457.68 eV (Ti2p_3/2_) and 463.41 eV (Ti2p_1/2_) respectively. The calculated ∆BE between Ti2p_3/2_ and Ti2p_1/2_ was 5.73 eV, which cannot be ascribed to the normal Ti^4+^ state in TiO_2_ and is an indication of the formation of Ti^3+^ species and/or mixtures of magnesium-titanium-oxygen Oxides Mg-Ti-O and calcium-titanium-oxygen Oxides Ca-Ti-O are formed with different oxidation states and/or stoichiometries, as demonstrated below with the X-ray diffraction results.

After mixing and heat treatment, the peak area of Ti2p_3/2_ has a BE of 457.68 eV, which is very close to 457.08 eV of Ti^3+^ in pure titania, increased by 6.17 times; similarly, the peak area of Ti2p_3/2_ in the mixture of brucite and titania has a BE of 459.28 eV, which is very close to 458.24 eV of the Ti^4+^ of pure titania, decreased by 99%. This suggests that the oxidation state present in the sample of brucite mixed with titania after heat treatment may correspond to Ti^4+^, but the difference of 0.6 eV may be due to the presence of mixtures of oxides with different stoichiometry. The change in stoichiometry was estimated by the change in area of relative peaks. The change from the Ti^3+^ peak area indicates that after brucite doping and heat treatment, oxygen is removed from the crystalline structure, showing a relative increase to Ti^3+^ in the XPS spectrum. On the other hand, with the decrease in the area of the Ti^4+^ new peak at 459.28 eV, the reaction of Mg^2+^ ion substitutions in the TiO_2_ crystalline structure can be inferred due to the reaction of Ti^4+^ ion substitutions in the MgO crystalline structure from the transformation of brucite at 1100 °C; on the other hand, mixtures of magnesium–titanium–oxygen oxides (Mg-Ti-O) and calcium–titanium–oxygen oxides (Ca-Ti-O) are formed with different oxidation states and/or stoichiometries, as demonstrated below with the X-ray diffraction results. This means that some mixed oxide structures are formed in large quantities, either with Mg or Ca, with a Ti^4+^ oxidation state after doping. Similarly, the new area of Ti^4+^ means that some mixed oxide structures are formed in large quantities, either with Mg or Ca, with a Ti^4+^ oxidation state after doping.

**Figure 6:**
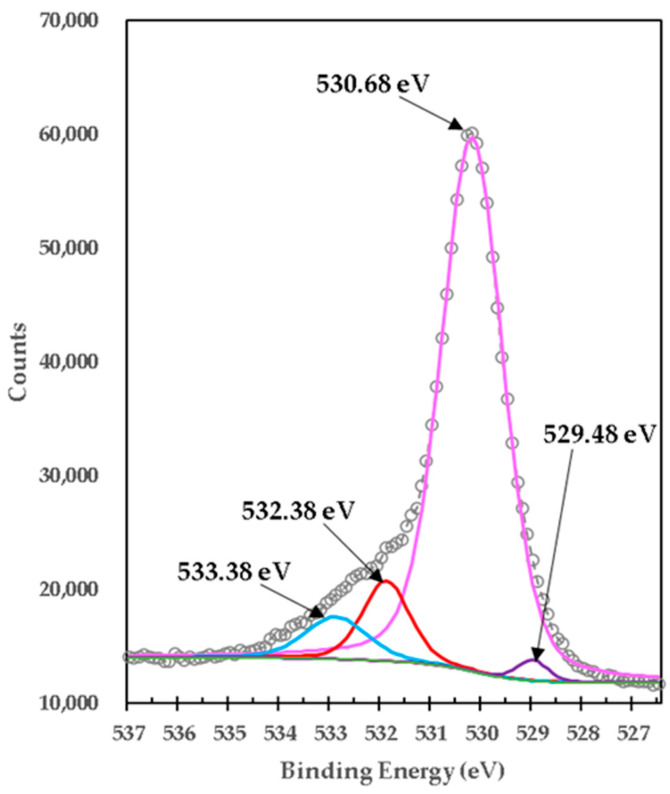
XPS high-resolution spectra of O1s in high-purity titania for formulation M3.

**Figure 7:**
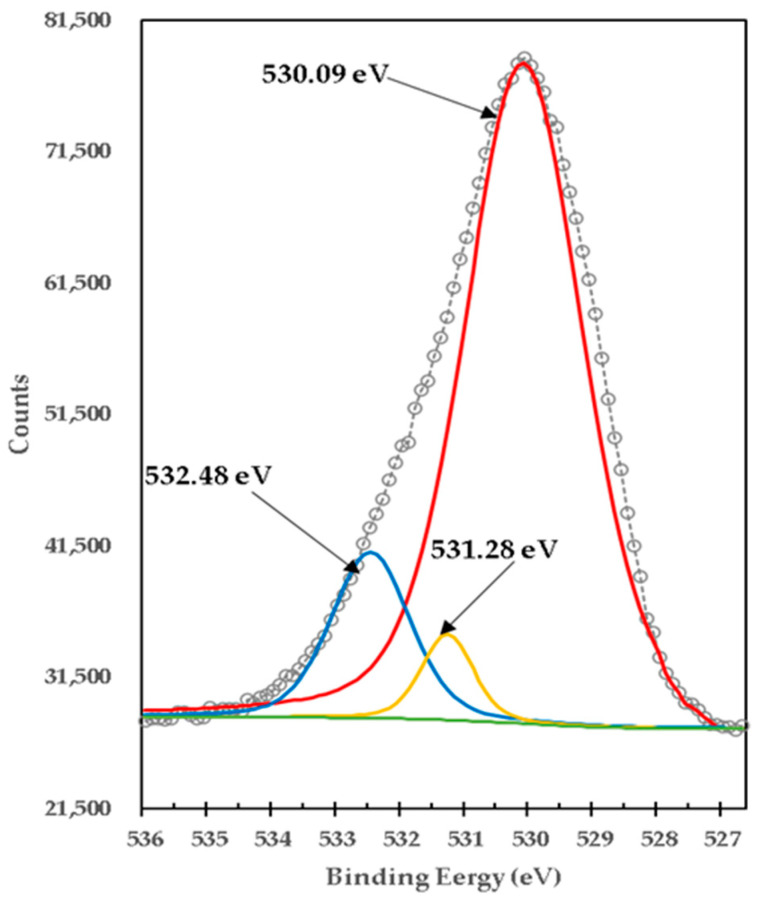
XPS high-resolution spectra of O1s in mixture of brucite and titania, calcined at 1100 °C for 1 h, for formulation M1.

Figure 6 shows the XPS high-resolution spectrum of O1s in high purity TiO_2_, which is composed of a peak at BE 530.68 eV, which was deconvoluted with three peaks located at 529.48 eV, 532.38 eV, and 533.38 eV. The highest binding energy at 533.38 eV is generally attributed to oxygen or hydroxyl (OH) species chemically absorbed or dissociated at the sample surface, such as adsorbed H_2_O [17]. The 532.38 eV bond energy component of O1s is associated with O_2_ ions found in the compound Ti_2_O_3_ [18], which is consistent with the XPS spectrum for Ti2p in Figure 7. The 529.48 eV bond energy component of O1s is associated with O^2−^ ions found in oxygen-deficient regions within the TiO_2_ matrix, promoted by the present chemical state of Ti^3+^. As a result, changes in the intensity of this component may be related to variations in the concentration of oxygen vacancies (VO) [19], which is consistent with the peak BE at 532.38 eV in the same spectrum by the chemical state of Ti^3+^, as well as with the XPS spectrum for Ti2p in Figure 4a. The peak intensity with a BE of 530.68 eV exceeds all other peaks, indicating the strong Ti-O binding in the pure TiO_2_ compound; this value is consistent with reference [14] and is further consistent with the XPS spectrum for Ti2p in Figure 4a. This indicates the formation of TiO_2_ and some mixed oxides.

Figure 7 shows the high-resolution XPS spectrum of O1s from the mixture of brucite with heat-treated titania. It consists of three peaks with BE of 530.09 eV, which in peaks with BE of 531.28 eV and 532.48 eV were deconvoluted. The calculated difference in BE of O1s (Figure 7) and Ti2p_3/2_ (Figure 5) (530.09 eV) − (457.68 eV) = 72.41 eV is in reasonable agreement with that of typical Ti^3+^ containing oxides (72.9 to 73.1 eV) [15]. The peaks at BE 457.68 eV (Ti2p_3/2_) and 463.41 eV (Ti2p_1/2_) were deconvoluted with two peaks located at 459.28 eV (Ti2p_3/2_) and 464.48 eV (Ti2p_1/2_) respectively, which can be attributed to the formation of Ti^4+^ species in mixtures of magnesium-titanium-oxygen Oxides Mg-Ti-O and calcium-titanium-oxygen Oxides Ca-Ti-O with different oxidation states and/or stoichiometries. Table 5 presents details on the data obtained and adjusted from XPS analysis of Ti2p for pure titania and brucite with titania mixture samples.

On other hand, Figure 7 shows the high-resolution XPS spectrum of O1s from the mixture of brucite with heat-treated titania. It was centered on BE of 530.09 eV. The peak at BE 530.09 was deconvoluted with two peaks located at 531.28 eV and 532.48 eV. The lowest bond energy of 530.09 eV corresponds to the strong bonds of O1s with different oxides of Ti [20]. The binding energy of 531.28 eV corresponds to O1s bonds with MgO [6,21]. The highest bond energy of 532.48 eV corresponds to both Mg(OH)_2_ [22] and different Ti oxides [12]. This indicates the formation of TiO_2_ and some mix oxides. All the binding energies of the XPS high-resolution spectra obtained for O1s in the samples of mixing TiO_2_ with brucite and heat-treated at 1100 °C for 1 h, show congruence with the phases determined in the X-ray diffractograms (XRD). Likewise, other authors have reported binding energies like those obtained in this work with respect to O1s in MgO. Gomez et al. found binding energies corresponding to ~531.40 eV and 531.73 eV, when MgO powders were compacted and sintered at 1550 and 1650 °C, respectively [23]. Pei Yun et al., also reported O1s in MgO at 530.05 eV, they obtained MgO/TiO_2_ compounds, through a suspension of magnesium carbonate hydroxide nanowires with ethanol and tetrabutyl titanate, the suspension was stirred, filtered, washed, dried, and calcined at 450 °C [24]. In another work, compacted and sintered Mg powder specimens were made at 600 °C for 40 min, the results in the binding energy of O1s for MgO of the specimens before sintering was 531.1 eV, and 532.2 after sintering [25]. Therefore, it is known that O1s peaks related to magnesium oxide have bending energies of approximately 530–531 eV, and magnesium hydrates have bending energies of approximately 530.0–533.2 eV [26,27,28,29,30]. Based on the previous works, the binding energies show shifts due to the procedures for obtaining each sample. In this work, the binding energy of O1s in MgO corresponds to 530.09 eV and O1s in Mg(OH)_2_ corresponds to 532.48 eV, which is close to those reported. From Figure 6 to Figure 7 it is observed that the peak with binding energy of 532.38 eV corresponding to Ti_2_O_3_ disappears after doping with brucite and heat treatment, but another very intense peak appears with binding energy of 530.09 eV corresponding to different types of Ti oxides, which indicates that in the process a mixture of different oxides between Ti^4+^ with Mg^2+^ and Ca^2+^ including TiO_2_ was formed. This is consistent with the phases identified in the X-ray diffraction diagrams. Again, the peak O1s binding energy of 530.68 eV corresponding to Ti^4+^ in pure titania shifts slightly to 530.09 eV after doping with brucite and heat treatment, which indicates that together with TiO_2_ a mixture of Mg-Ti-O oxides and Ca-Ti-O oxides are formed. The change in stoichiometry is estimated by the change in area of relative peaks. In this case, the O1s peak of 532.38 eV corresponding to Ti^3+^ in pure titania shifts slightly to 530.09 eV, which corresponds to a mixture of different oxidation states, and increases its area by 17 times. Doping with Mg^2+^ ions by brucite results in a minor change in binding energy, indicating that Mg^2+^ ions are better dispersed at TiO_2_ crystalline structure substitution sites and produce more Mg-O-Ti mixed oxide structure, which is consistent with the results of phases identified with X-ray diffraction. Table 6 presents details on the data obtained and adjusted from the XPS analysis of O1s for pure titania and brucite-with-titania mixture samples.

**Figure 8:**
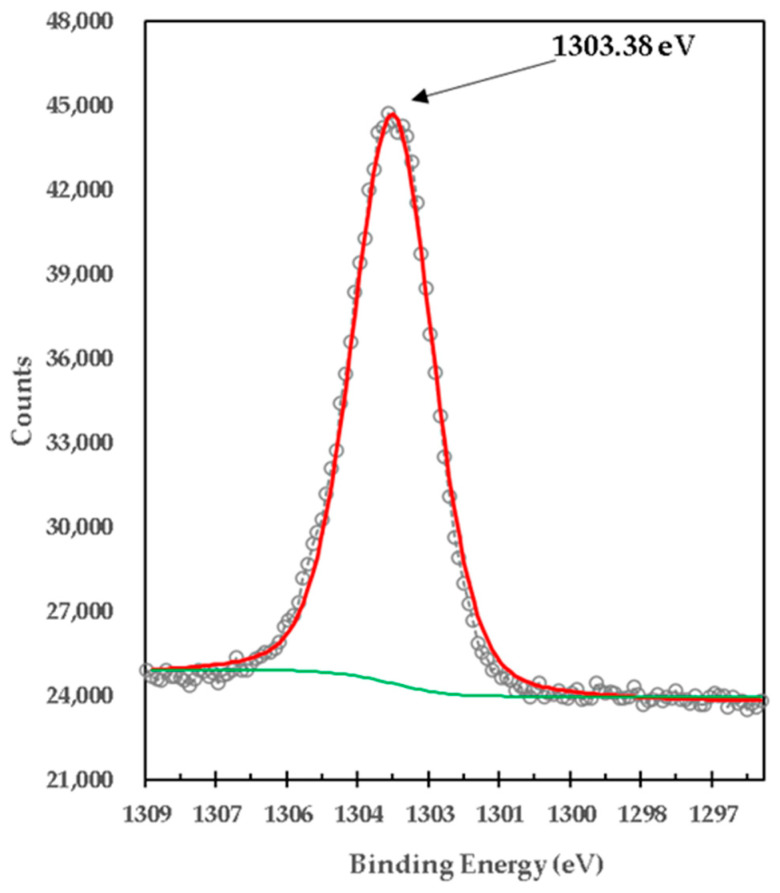
XPS high resolution spectra of Mg1s in brucite M4.

**Figure 9:**
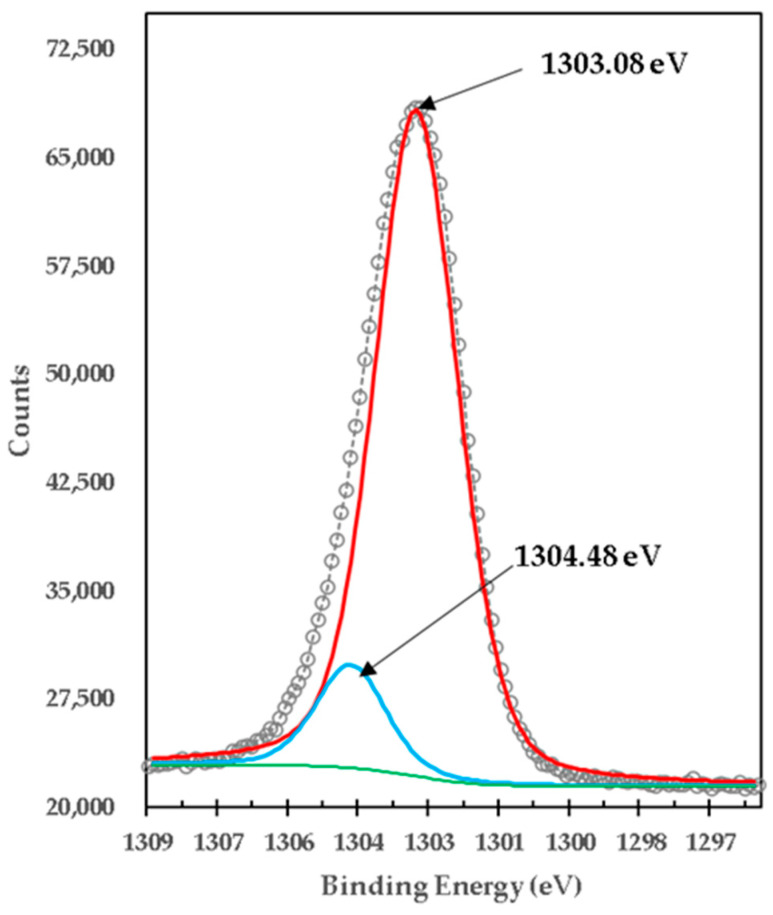
XPS high-resolution spectra of Mg1s in calcined brucite at 1100 °C for 1 h M2.

Figure 8 presents the XPS high-resolution spectrum of Mg1s of brucite without doping with TiO_2_ and without heat treatment. It consists of a single peak with a binding energy of 1302.69 eV, which corresponds to the brucite compound, Mg(OH)_2_ [31]; this measurement is consistent with X-ray diffraction results. To characterize the brucite and analyze its transformation when comparing against the mixture, brucite was calcined at 1100 °C for 1 h without doping with TiO_2_. Figure 9 presents the XPS high-resolution spectrum of Mg1s for calcined brucite. It consists of two peaks with BE of 1303.08 eV and 1304.48 eV, which correspond to the compounds of MgO and Mg(OH)_2_, respectively, corresponding to Mg^2+^ [32]; this is consistent with X-ray diffraction results. 

**Figure 10:**
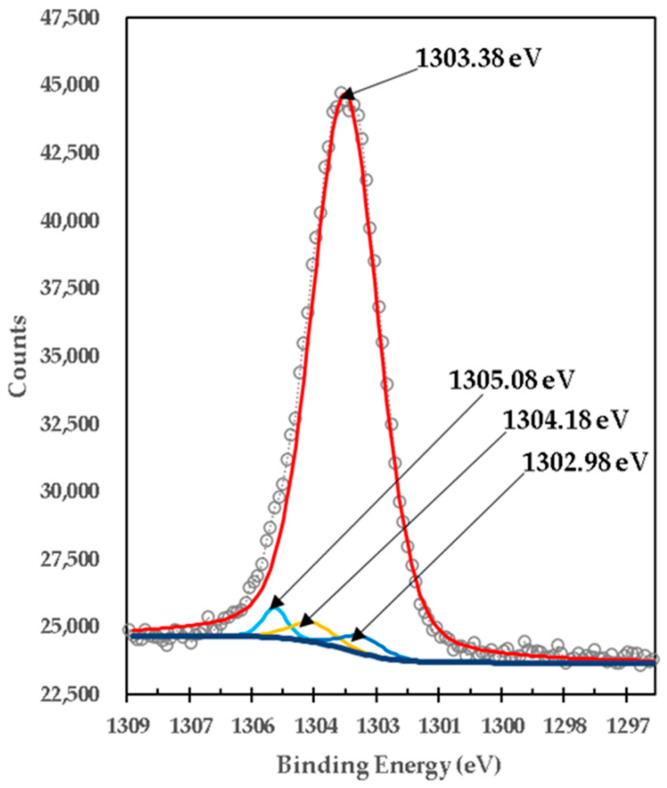
XPS high-resolution spectra of Mg1s in titania mixed with brucite and calcined at 1100 °C for 1 h M1.

Figure 10 presents the XPS high-resolution spectrum of Mg1s of the mixture of brucite with heat-treated titania. It was centered in one peak with a BE of 1303.38 eV, which was deconvoluted with three peaks located at 1302.98 eV, 1304.18 eV and 1305.08 eV. The lowest bond energy of 1302.98 eV is very close to the 1302.69 eV obtained from uncalcined brucite shown in Figure 8, so it is attributed to the strong bonds of Mg1s electrons in the brucite compound, Mg(OH)_2_ [31]. It is observed that its area is reduced by 98.42% from 122,078.39 CPS to 1917.12 CPS, which is consistent with the X-ray diffraction results.

The binding energy of 1303.38 eV, according to its high peak area corresponding to the percentage obtained in X-ray diffraction analyses, is attributed to the strong bonds of Mg1s electrons in the compound MgTi_2_O_5_. The binding energy of 1304.18 eV is very close to the 1304.30 eV obtained from the calcined brucite shown in Figure 9, so it is attributed to the strong bonds of the Mg1s electrons in the magnesia compound, MgO, corresponding to Mg^2+^ [32], which is consistent with the X-ray Diffraction results. The binding energy of 1305.08 eV, according to its peak area corresponding to the percentage obtained in X-ray diffraction analyses, is attributed to the strong bonds of Mg1s electrons in the MgTiO_3_ compound.

**Figure 11:**
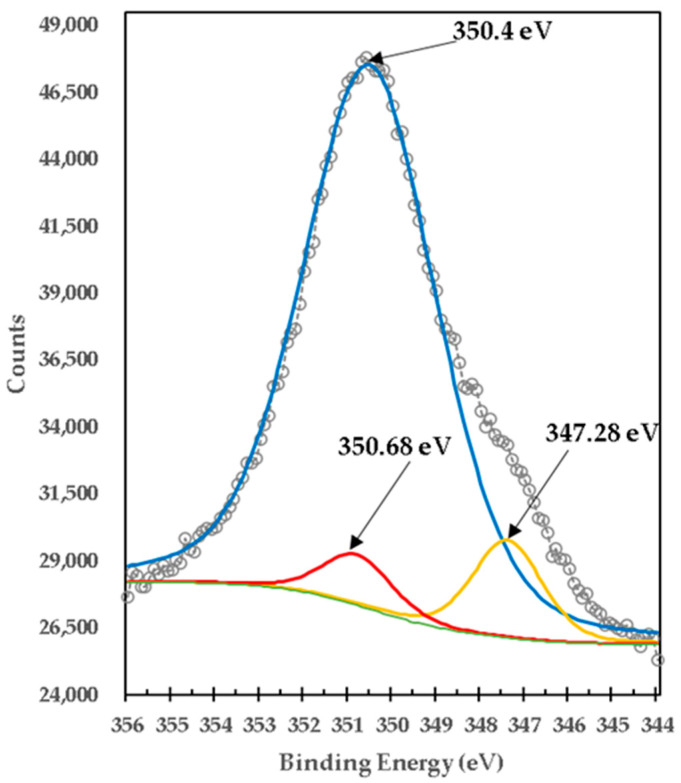
XPS high-resolution spectrum of Ca2p in titania mixed with brucite and calcined at 1100 °C for 1 h M1.

**Figure 12:**
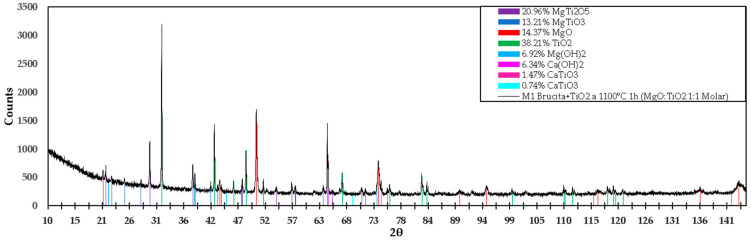
X-ray diffractogram of the formulation M1 (titania mixed with brucite and calcined at 1100 °C for 1 h).

Figure 11 presents the high-resolution XPS spectrum of Ca2p of the mixture of brucite with heat-treated titania. One peak was centered on BE of 350.4 eV, which was deconvoluted with a double peak located at 347.28 eV (Ca2p_3/2_) with 350.68 eV (Ca2p_1/2_) that could be due to Ca(OH)_2_ and mix of CaTiO_3_ [33,34], demonstrated by the X-RD analysis shown in Figure 12.

### 3.2. Microstructural Analysis by XRD

The diffractogram in Figure 12 shows the different compounds detected in the M1 mixture. For the compound MgTi_2_O_5_ with a percentage by weight of 20.96%, angles of 21.173°, 29.662°, 37.964°, 43.543°, 44.116°, 54.134°, 57.088°, 65.684°, 70.625°, and 71.346° were detected, of which the diffraction planes are (200), (220), (230), (131), (311), (430), (002), (222), (232), and (531), respectively; these coincide with the values of the reference ICDD 04-009-8048. For the MgTiO_3_ compound with a percentage by weight of 13.21%, the matching angles are 27.954° and 57.769°, and the diffraction planes are (012) and (024), respectively, in agreement with PDF reference 01-080-2548. A percentage by weight of 14.37% MgO was detected, and the angles referenced as MgO are 43.174°, 50.279°, 73.852°, 89.578°, 94.749°, 116.346°, and 143.567°; the diffraction planes are as follows: (111), (200), (220), (311), (222), (400), (420), respectively, in agreement with reference ICDD 04-004-8990.

For TiO_2_ with the highest percentage by weight of 38.21%, its angles coincide with 31.980°, 42.157°, 45.854°, 48.283°, 51.642°, 64.038°, 66.863°, 76.044°, 83.275°, 99.743°, 111.463°, 118.212°, 119.330°, and 121.167°, with diffraction planes (110), (101), (200), (111), (210), (211), (310), (112), (321), (330), (411), (312), and (420), respectively, in agreement with the values referenced in ICDD 04-008-4342. Mg(OH)_2_ has a percentage by weight of 6.92%, and the angles obtained in the diffractogram coincide with the values of the reference ICDD 04-016-3445, whose angles are 38.320°, 73.547°, 119.691°, and 142.158°, with diffraction planes (100), (111), (023), and (122), respectively.

Ca(OH)_2_ is present at 6.34% by weight in the M1 mixture, which can be verified since the angles obtained coincide with the reference ICDD 01-076-0570. The angles are 21.124°, 74.382°, and 115.478°, with the following diffraction planes: (001), (201) and (122), respectively. The CaTiO_3_ compound is present at 1.47% by weight; its angles are referenced with ICDD: 86-1393, whose congruent angle is 47.5° and diffraction plane is (220); additionally, in agreement with reference ICDD 98-003-7263, the corresponding angles are 47.5° and 69.4° and the corresponding diffraction planes are (040) and (242), respectively.

**Figure 13:**
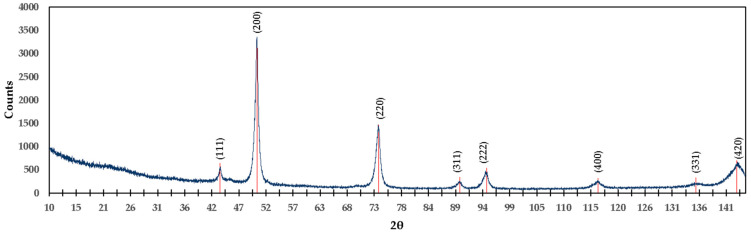
Diffractogram of the M2 formulation (calcined brucite at 1100 °C 1 h), according to ICDD 04-004-8990.

Figure 13 presents the XRD diagram of the mixture M2 (calcined brucite), which resulted in caustic MgO. The planes obtained in X-ray diffraction are as follows: (111), (200), (220), (311), (222), (400), (331), and (420), corresponding to angles 43.174°, 50.279°, 73.852°, 89.578°, 94.749°, 116.346°, 135.607°, and 143.567°, respectively, in agreement with reference ICDD 04-004-8990. By comparing this information with the XPS analysis, it can be concluded that MgO is present in this formulation.

**Figure 14:**
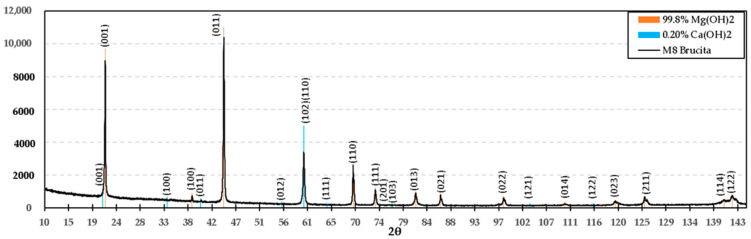
Diffractogram of the M4 formulation (uncalcined brucite).

Brucite was used in the formulation of M4; its diffractogram is shown in Figure 14. Two compounds were determined in this sample, and the planes obtained from Mg(OH)_2_ are as follows: (001), (100), (011), (102), (110), (111), (013), (021), (022), (014), (023), and (211), corresponding to angles 21.626°, 38.320°, 44.426°, 59.801°, 68.503°, 69.289°, 73.547°, 81.316°, 82.055°, 86.114°, 97.254°, 98.238°, 106.251°, 109.983°, 119.691°, 120.539°, 125.340°, 139.446°, 140.605°, 142.158°, 159.860°, 167.217°, and 171.116°, respectively, in agreement with reference ICDD 04-016-3445.

For Ca(OH)_2_, which is present at only 0.20% by weight, the following diffraction planes were detected: (001), (100), (011), (012), (110), (111), (201), (103), (121), and (122), corresponding to angles 21.124°, 33.479°, 39.925°, 43.012°, 55.576°, 59.849°, 64.209°, 66.719°, 70.343°, 74.382°, 76.498°, 76.742°, 86.122°, 94.311°, 95.881°, 99.281°, 103.212°, 103.941°, 105.573°, 115.478°, 119.558°, 124.083°, 124.945°, 132.84°, 137.611°, 140.004°, 147.766°, and 172.165°, respectively, in agreement with reference ICDD 01-076-0570. Comparing these compounds with the XPS results, we can confirm that in the M8 formulation, the compound Ca(OH)_2_ is present.

**Figure 15:**
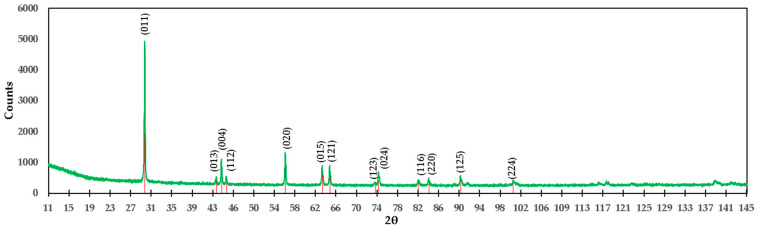
Diffractogram of the M3 formulation (high-purity titania), showing the identification of TiO_2_.

**Figure 16:**
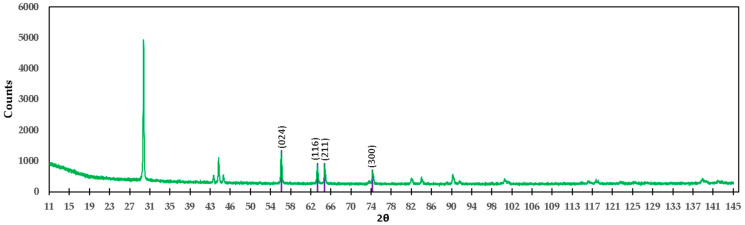
Diffractogram of the M3 formulation (titania of high purity), showing the identification of Ti_2_O_3_.

Figure 15 shows the diffractogram of TiO_2_. According to this diagram, in the mixture of the M3 formulation, the compound TiO_2_ is present. In the same diffractogram, the planes with respective angles 2θ belonging to this compound are shown to be in agreement with ICDD 04-008-4342, which supports the presence of TiO_2_. For the M3 formulation, the presence of the compound Ti_2_O_3_ was also found; the diffractogram of this compound is shown in Figure 16.

**Figure 17:**
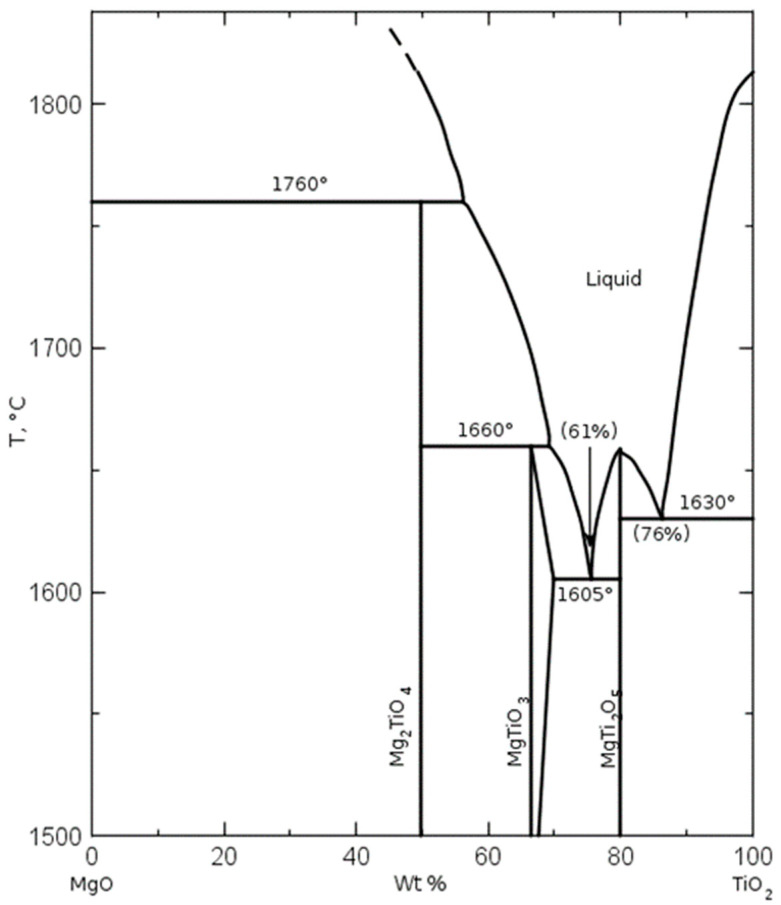
Phase diagram of MgO and TiO_2_ showing the phases formed with different contents of the substances at different temperatures [35].

As can be seen in the diffractograms and in the XPS spectra, it is confirmed that by mixing TiO_2_ with industrial-grade brucite of national origin and calcining at a low temperature (1100 °C) for a short period of time (1 h), it is possible to form ceramic phases mainly in the Mg-Ti-O system. The compounds that are obtained are mainly MgTi_2_O_5_, MgTiO_3_, MgO, and TiO_2_. The percentages obtained are relatively high: approximately 21, 13, 14, and 38% for MgTi_2_O_5_, MgTiO_3_, MgO, and TiO_2_, respectively. When analyzing the phase diagram in Figure 17, the compounds between MgTi_2_O_5_ and MgTiO_3_ have melting points between 1605 °C and 1660 °C.

Considering that MgO-based refractory materials produced from double-calcined brucite are used in melting processes with temperatures above 1537 °C in steel production, for example, it is appropriate to take care of the formation of these compounds during the sintering of MgO if it is doped with titanium ions by micro or TiO_2_ microparticles.

Additionally, Table 7 shows that the reticular values of the titania and magnesium titanate phases are exactly consistent with the values of the crystal structure of these compounds, as well as the MgO lattice parameters in the calcined brucite (M2) sample; on the other hand, it is observed that for the reticular values of MgO in the mixture of brucite with heat-treated titania at a temperature of 1100 °C for 1 h (M1), the crystallographic parameters of MgO are modified as a result of the presence of Ti ions in its crystal structure and possible vacancies generated by the greater number of valence electrons between Mg and Ti.

## 4. Conclusions

It is concluded that the incorporation of Ti^4+^ ions by mixing TiO_2_ microparticles in the brucite slightly modifies the crystallographic structure of the caustic MgO obtained after its calcination at 1100 °C 1 h, forming compounds of the Mg-Ti-O system. In addition, it is concluded that Ti^4+^ modifies the size of the crystal structure, possibly due to the demand of twice as many O ions- generating vacancies in the crystal structure.

It is concluded that the presence of TiO_2_ in brucite promotes the formation of MgTi_2_O_5_ and MgTiO_3_ compounds, which have relatively low melting points; care must be taken during the addition of said oxides in the densification of the double calcined MgO. Additionally, it is concluded that the low oxidation states in TiO_2_ generate oxygen vacancies in the crystal lattice structure. Finally, it is concluded that, of the impurities of Ca, Fe, Al, and Si in the Mexican brucite, only the presence of Ca influences calcination in the interaction of TiO_2_, forming CaTiO_3_ compounds.

## Figures and Tables

**Table 1:** Chemical composition of the brucite produced from synthetic brine in Mexico used in the present work.

MgO (% Weight)	CaO (% Weight)	SiO_2_ (% Weight)	Fe_2_O_3_ (% Weight)	Al_2_O_3_ (% Weight)	LOI (% Weight)
46.00	0.31	0.04	0.02	0.04	53.59

**Table 2:** Percentage by weight for brucite- and titania-based formulations.

Compound	Molar Weight gr	% Weight
Mg(OH)_2_	58.3197	42.20
TiO_2_	79.8658	57.80
Total	138.1855	100

**Table 3:** List of formulations developed.

	Mg(OH)_2_ % Mol	TiO_2_ % Mol	Calcined Brucite
M4 (not calcined)	100	0	0
M3 (not calcined)	0	100	0
M2 (calcined)	0	0	100
M1 (calcined)	50	50	0

**Table 4:** Data from XPS measurements on the pure titania used in this work and calculations to determine the precentage of oxygen vacancies in the crystal lattice.

Ion	Peak Binding Energy eV	FWHM eV	Area CPS eV	% Ti^3+^	% Ti^4+^	O/Ti	% O	% Vacancies of O
Ti^4+^	•458.53	1.87	58,355.26	4%	96%	1.979966759	99%	1%
Ti^3+^	•457.08	0.62	2338.09					

**Table 5:** Details of the data obtained from the XPS analysis of Ti for samples of pure titania and mixture of brucite with titania, with the latter heat treated at 1100 °C for 1 h.

Sample	Ion	Peak Binding Energy eV	Area CPS eV	Area Ratio	FWHM eV
High-purity titania	•Ti^4+^	458.24	63,407.41	1	1.27
	•Ti^4+^	463.68	30,579.04	0.48	2.18
	•Ti^3+^	457.08	3991	0.06	0.84
	•Ti^3+^	463.28	2056.9	0.03	0.84
Brucite + TiO_2_ mixture calcined at 1100 °C 1 h (1:1 Molar MgO:TiO_2_)	•Ti^3+^	457.68	20,655.55	1	1.29
	•Ti^3+^	463.41	9098.07	0.44	2.04
	•Ti^4+^	459.28	465.95	0.02	0.46
	•Ti^4+^	464.48	730.39	0.04	0.52

**Table 6:** Details of the data obtained from XPS analysis of O1s for samples of pure titania and mixture of brucite and titania, with the latter heat treated at 1100 °C for 1 h.

Sample	Ion	Peak Bond Energy Ev	Area CPS Ev	Area Ratio	FWHM Tight Ev
High-purity titania	•O1s (TiO_2_)	530.68	80,898.97	1	1.45
	•O1s (O_2_ deficiencies in TiO_2_)	529.48	1592.77	0.02	0.7
	•O1s (Ti_2_O_3_)	532.38	9340.62	0.12	1.1
	•O1s (OH)	533.38	6457.01	0.08	1.46
Brucite + TiO_2_ mixture treated at 1100 °C 1 h (1:1 Molar MgO:TiO_2_)	•O1s (Varied Ti compounds)	530.09	167,660.41	1	2.9
	•O1s (MgO)	531.28	121.15	0.00	0.94
	•O1s (varied compounds Mg-Ti-O)	532.48	9048.83	0.05	1.46

**Table 7:** Cristallographic parameters obtained from XRD analysis for samples of pure calcined brucite (M2) and brucite mixed with titania (M1), both heat-treated at 1100 °C for 1 h.

Sample	Compound	Crystal Structure	Space Group	*a* Å	*b* Å	*c* Å	α °	β °	γ °
M2	MgO	Cubic	F m −3 m (225)	4.213313	4.213313	4.213313	90	90	90
M1	MgO	Cubic	F m −3 m (225)	4.21156	4.21156	4.21156	90	90	90
	MgTiO_3_	Rhombohedral	R −3 (148)	5.0549	5.0549	13.8939	90	90	90
	MgTi_2_O_5_	Orthorhombic	C m c m (63)	3.7428	9.7387	9.9976	90	90	90
	TiO_2_	Tetragonal	P 42/m n m (136)	4.59327	4.59327	2.95892	90	90	90

## Data Availability

Not applicable.
